# Supplementary material for: Genome-wide identiffcation of the WRKY gene family in poplar and the positive role of PsnWRKY95 in response to cadmium stress
Source: PLoS One. 2025 Sep 12;20(9):e0332076. doi: 10.1371/journal.pone.0332076 (PMC12431293; doi:10.1371/journal.pone.0332076)
Supplement: Attachment — Submitted filename: Response to Reviewers.doc [file pone.0332076.s015.doc]

Dear Editor,

Thank you for your letter and the comments concerning our manuscript entitled “Genome-Wide Identiffcation of the WRKY Gene Family in Poplar and the Positive Role of PsnWRKY95 in Response to Cadmium Stress" (Manuscript ID: PONE-D-25-02876). Those comments are all valuable and very helpful for revising and improving our paper, as well as the important guiding significance to our researches. We have studied comments carefully and have made correction which we hope meet with approval. Revised portions are marked in yellow in the paper. The main corrections in the paper and the responses to the reviewer's comments are as follows:

**Reviewer #1:**

1. **Response to comment:**

I suggest incorporating a graphical abstract as well as an abbreviation list;

**Response:**

Thank you for your valuable advice. We have included the graphical abstract and abbreviation list in line35-line41 of the manuscript.

2. **Response to comment:**

The abstract could be improved by providing more quantitative information regarding the obtained results and greater specificity in the details. For example, the authors mention improvements in growth parameters and Cd tolerance upon regulation mediated by PsnWRKY95 but do not provide specific details about these aspects;

**Response:**

Thank you for your valuable advice. We have included specific details about the growth parameters, cadmium tolerance and quantitative information indicators of PsnWRKY95 transgenic poplars in the abstract.

3. **Response to comment:**

It would be beneficial to cite more recently published references from 2024 and 2025 throughout the manuscript, including in the introduction;

**Response:**

Thank you for your careful work. We have incorporated the latest relevant research findings from 2024-2025 into the introduction, such as references 5-8 and 28.

4. **Response to comment:**

The introduction could be improved by better separating paragraphs when introducing new ideas—for instance, the introduction of transcription factors (line 53) could begin in a separate paragraph.

**Response:**

Thank you for your valuable advice. We have made the introduction of transcription factors has been placed in a separate paragraph.

5. **Response to comment:**

Additionally, a better contextualization of the biochemical approaches used in the study would help readers understand the methodological expectations, particularly regarding antioxidant molecules;

**Response:**

Thank you for your careful work. We have included the specific biochemical methods in Section 2.9, line 248.

6. **Response to comment:**

In line 110, the authors state that "Resistance to heavy metal adversity is crucial for plant survival," but I wonder whether, under real-life conditions, heavy metals actually cause plant death or significant productivity loss in agricultural settings. Personally, I am not aware of major yield losses caused by heavy metals compared to other abiotic factors such as drought, salinity, or extreme temperatures;

**Response:**

Thank you for your careful work. Numerous studies show heavy metals cause plant death or reduced agricultural productivity. Heavy metal toxicity (HMT) threatens global agriculture, impairing crop yields via physiological damage, reduced germination, oxidative stress, and inhibited photosynthesis. Cadmium (Cd), accumulating in plants with a 10-30 year half-life, induces root browning, growth inhibition (reduced length/dry weight, impaired lateral roots) and death; it also exacerbates growth inhibition/necrosis by disrupting carbon fixation, chlorophyll levels and photosynthesis, and triggers excessive reactive oxygen species (ROS) via antioxidant system disruption, damaging cells and plants. Soil Cd pollution further reduces produce quality, endangers human health, and undermines agricultural sustainability. We have supplemented this information in line 114 of the introduction.

7. **Response to comment:**

Regarding the methodology, details about the selection of plant material, cadmium concentration in the growth medium, and exposure duration to the heavy metal are not clearly provided;

**Response:**

Thank you for your careful work. Regarding the details of this part of the experimental methods, we have provided a detailed explanation in Section 2.5, line 189.

8. **Response to comment:**

For the results section, I suggest incorporating better organization through tables or figures summarizing the main expression patterns and phylogenetic relationships, which could facilitate data interpretation. Additionally, including real photos and figures from the experiments would enhance transparency for readers;

**Response:**

Thank you for your valuable advice. Due to the large number of members in the WRKY family genes, the tables or figures illustrating the main expression patterns and phylogenetic relationships are too large to be included in the main text, so they have been placed in the supplementary materials. Additionally, Fig 2, 3, 5, 8, and 9 have been re-adjusted to enhance transparency for readers.

9. **Response to comment:**

Importantly, there are other studies closely related to this research topic, also focusing on poplar and cadmium stress. For example:

• "A WRKY transcription factor, PyWRKY75, enhanced cadmium accumulation and tolerance in poplar" - Ecotoxicol Environ Saf. 2022 Jul 1;239:113630. doi: 10.1016/j.ecoenv.2022.113630

• "A WRKY transcription factor, PyWRKY71, increased the activities of antioxidant enzymes and promoted the accumulation of cadmium in poplar" - Plant Physiol Biochem. 2023 Dec;205:108163. doi: 10.1016/j.plaphy.2023.108163

It is unclear why the authors did not consider these studies for a more comparative discussion on WRKY, poplar, and cadmium-related findings. Such a comparative discussion would also help better contextualize the novelty of the current study compared to previous ones;

**Response:**

Thank you for your careful work. This is an excellent and insightful point. These two reports offer a more comprehensive and specific perspective on the research of poplar under cadmium stress, with much to learn from. We have incorporated the findings of these two studies into the discussion section of our research, specifically in line 646.

10. **Response to comment:**

The discussion could provide a more critical analysis of the study’s limitations, particularly regarding the methodologies used to validate PsnWRKY95’s role in cadmium tolerance and gene expression results. Additionally, it would be relevant to highlight the implications of introducing an exogenous gene under real agricultural conditions;

**Response:**

Thank you for your valuable advice. By referring to peer research methods on cadmium stress in poplars, we have gained significant insights and recognized the limitations of our research methods. We have analyzed these methodology in the discussion section. Additionally, we have analyzed the benefits of introducing exogenous genes in practical agricultural applications, as detailed in line 653.

11. **Response to comment:**

Conclusion section: The conclusion broadly summarizes the study’s approaches but lacks reflection on the practical implications of the research, as well as a more specific discussion of the obtained quantitative results and insights.

**Response:**

Thank you for your careful work. We have revised the conclusion to include practical implications, specific quantitative results, and key insights as suggested, in line 674.

**Reviewer #2:**

1. **Response to comment:**

Many figures are large with very small fonts, making them not very comprehensive for someone reading in a pdf file.

**Response:**

Thank you for your careful work. We have revised the figures, particularly Fig 3, 5, 8, and 9.

2. **Response to comment:**

Many abbreviations were not defined upon first appearance in the text, some were not given, others only late in the text (eg. SA was defined in line 565 and used in line 93).

**Response:**

Thank you for your careful work. We have provided comprehensive explanations of abbreviations and included an abbreviation list in manuscript.

3. **Response to comment:**

There is some confusion with the names PtWRKY and PsnWRKY: The systematic analysis of physicochemical properties, phylogenetics, gene structure, chromosome distribution, evolutionary relationship, promoter cis-acting elements, GO functional annotation, target miRNA and STRING protein-protein interaction network were all made using the ensembl available poplar genome that is from *Populus trichocarpa* (Pt) while the expression analysis (RNA-Seq, RT-qPCR), cloning, subcellular localization and overexpression in tobacco were using the hybrid *Populus simonii × P. nigra* (Psn) genetic background. In some parts of the text they are treated as the same (eg., expression analyses), in others mixed (e.g., STRING analysis). A simple explanation in the text could make the distinction clear, and if there are such low sequence distinctions, in some sections it would be better to just address the genes without a specific suffix, just as WRKY.

**Response:**

Thank you for your careful work. The main experimental subject of this study is *Populus simonii × P. nigra*. Compared with other poplar varieties, *Populus simonii × P. nigra* has the characteristics of fast growth, strong adaptability and good cold resistance, and can grow in high-cold regions such as Heilongjiang, China. Through mass selection of improved varieties, regional trials and years of practice, it has been proved to be an excellent fast-growing and high-yielding tree species. However, the genome of *Populus simonii × P. nigra* is still incomplete and has not been published. *Populus trichocarpa*, as a model plant among woody plants, is a typical representative tree species for studying forest genetics. Its genome was sequenced and published in 2006, and has been repeatedly corrected, which is relatively mature and effective. We have carried out a large amount of transcriptome sequencing work on *Populus simonii × P. nigra*. Through comparison, it was found that the gene sequences of *Populus simonii × P. nigra* and *Populus trichocarpa* are extremely similar with high homology, both reaching more than 99%. Therefore, in this study, the *Populus trichocarpa* genome was used as the reference genome to search for genes, and systematic analyses of physicochemical properties, phylogenetics, gene structure, chromosome distribution, evolutionary relationship, promoter cis-acting elements, GO functional annotation and target miRNA were performed. The expression analysis, cloning, subcellular localization, STRING protein-protein interaction network and overexpression in tobacco were conducted using the hybrid *Populus simonii × P. nigra* genetic background. Among them, the STRING protein-protein interaction network analysis was performed using the PsnWRKY95 sequence obtained by cloning and sequencing. We have corrected the text in Section 2.6 and Figure 10. In addition, the information about the two poplar species mentioned above has been explained in the introduction and Section 2.1 of the methods.

4. **Response to comment:**

By the way, both expression experiments were made with RNA-seq? It was not clear to me for the “temporal” experiment if there were only 12 genes between all 102 that showed the distinction, or that these were previously chosen.

**Response:**

Thank you for your careful work. Both expression experiments were conducted RNA-seq. From the results of the spatiotemporal expression pattern experiment, it was found that the majority of WRKY genes respond to cadmium stress. However, presenting the results of all members would make the manuscript excessively lengthy. Therefore, only 12 genes with higher response levels were selected for result presentation. The selection was based on the results of the significant differential expression experiment, 12 genes, including WRKY4, WRKY19, and WRKY38 showed the most significant results.

5. **Response to comment:**

I had this discussion with colleagues before, and you like the authors’ opinion, could not the GFP-tag in the cloned protein C-terminal end affect the function of the studied protein? Could this be relevant to the observed results?

**Response:**

Thank you for your careful work. We also had concerns about this issue when we initially conducted the transgenic research. However, based on the research results from myself and colleagues in our group, who have performed genetic transformation and expression experiments of other genes in poplar, the results consistently showed that the GFP tag at the C-terminus of the cloned protein hardly affects the function and phenotype of the target protein. The relevant research findings are as follows:

Guo Q, Wei R, Xu M, Yao W, Jiang J, Ma X, Qu G, Jiang T. Genome-wide analysis of HSF family and overexpression of PsnHSF21 confers salt tolerance in Populus simonii × P. nigra. Front Plant Sci. 2023 Apr 26;14:1160102.

Cheng Z, Zhu Y, He X, Fan G, Jiang J, Jiang T, Zhang X. Transcription factor PagERF110 inhibits leaf development by direct regulating PagHB16 in poplar. Plant Sci. 2025 Jan;350:112309.

Cheng Z, Fan G, Jiang J,et al.Transcription factor PagERF110 inhibits xylem differentiation by direct regulating PagXND1d in poplar[J].Industrial Crops and Products, 2024, 215(000):14.

6. **Response to comment:**

And just another question, just for curiosity, the poplar trees that become more resistant to heavy metal contaminated soil would be bioaccumulators? And how could the accumulated metal affect the destination of these trees? Could the wood, coal or paper be used in the same manner as the “normal” poplar?

**Response:**

Thank you for your careful work. Yes, poplar can act as bioaccumulators of heavy metals in soil. Numerous studies have shown that the regulation of related genes in plant transgenic technology can lead to the accumulation of heavy metals in different tissues of trees. For example, PyWRKY75 significantly enhances the cadmium uptake and accumulation capacity of poplars, with the cadmium uptake of the OE-41 line being 51.32% higher than that of the wild type (Xiaolu W, Qi C, Lulu C, et al. A WRKY transcription factor, PyWRKY75, enhanced cadmium accumulation and tolerance in poplar. [J]. Ecotoxicology and Environmental Safety, 2022, 239: 113630). PyWRKY71 significantly promotes cadmium accumulation in poplars, especially in roots the cadmium content in the OE-45 and OE-87 lines reaches 1.42 times that of the wild type (Xiaoxi C, Xiaolu W, Chengyu H, et al. A WRKY transcription factor, PyWRKY71, increased the activities of antioxidant enzymes and promoted the accumulation of cadmium in poplar. [J]. Plant Physiology and Biochemistry : PPB, 2023, 205: 108163). In rice, OsHMA3 sequesters cadmium into the vacuoles of root cells, restricting cadmium transport from roots to shoots and reducing cadmium accumulation in grains; in mutants, cadmium accumulation in roots decreases, while cadmium content in grains increases (Lu C, Zhang L, Tang Z, Huang XY, Ma JF, Zhao FJ. Producing cadmium-free Indica rice by overexpressing OsHMA3. Environ Int. 2019 May; 126: 619-626; Yan H, Jiao X, Chen Y, et al. Knockout of OsHMA3 in an indica rice increases cadmium sensitivity and inhibits plant growth [J]. Plant Growth Regulation, 2024, 103(3): 635-646).

As for whether the wood, coal, or paper derived from these poplars can be used in the same manner as those from "normal" poplars, we are currently conducting poplar transformation of PsnWRKY95, and the these issues will be further studied in subsequent work.

7. **Response to comment:**

More punctual questions and observations can be seen in the pdf file with my comments.

**Response:**

Thank you for your careful work. We have revised the issues marked in the manuscript PDF.

**Reviewer #3:**

1. **Response to comment:**

One of my main concern is related to the extensive bioinformatics analysis, which overlaps with previous works, as I mention below, that are not mentioned or contrasted by the authors. There is no clear reason to redo all these analyses and I do not see how these results are novel and better than those published.

There are previous works with WRKY TFs in Poplar. He et al. (2012, doi: 10.1007/s00299-012-1241-0) identified 104 WRKY genes. How is the classification in this MS better than that of He et al. Why a new phylogeny is needed and how do they compare. ¿Are gene IDs and names related or linked?

In another work, Chen et al. (2022, doi: 10.3390/genes13122324) presented an analysis of the WRKY family in Poplar, also proposing a phylogeny and studying gene location, collinearity, protein motifs, gene structure and cis-acting elements. How is the new informatio better than this, why is a new analysis relevant or necessary?

**Response:**

Thank you for your careful work. Since 2012, significant progress has been made in genome sequencing technologies, and high-quality poplar genomes have undergone multiple revisions. He et al. (2012, doi: 10.1007/s00299-012-1241-0) identified 104 WRKY genes in 2012. Our study, based on the latest genome database (*Populus_trichocarpa*.Pop_tri_v4.61 from Ensembl Plants) and using strict criteria to exclude pseudogenes, identified 102 WRKY genes. The difference between the two studies is only 2 genes (approximately 1.9%), which is a common phenomenon in gene family identification research. This discrepancy mainly stems from two aspects: first, the use of different versions of poplar genome assembly/annotation (version updates will optimize gene models, leading to minor adjustments in the number of members); second, differences in the details of identification criteria, such as varying requirements for the integrity of the WRKY domain and different handling methods regarding whether pseudogenes are included in the statistics. Importantly, both results confirm that poplars possess a large and complex WRKY family, and this difference does not affect the core conclusions (phylogenetic analysis, structural analysis, expression analysis, etc.). The gene naming in both studies starts from the first gene on the first chromosome; except for the 2 excluded pseudogenes, the names of other genes are consistent. Regarding the gene IDs in the two studies, POPTR_X and Potri.X are two different numbering systems, where the former is a species identification number and the latter is a species abbreviation number.

In another study, Chen et al. (2022, doi: 10.3390/genes13122324) presented an analysis of the WRKY family in poplars, including phylogenetic analysis, gene location, collinearity, protein motifs, gene structure, and cis-acting elements, but their research was conducted in the context of poplar WRKY genes regulating nitrogen nutrition. Our research focuses more on poplar WRKY genes in response to cadmium stress. In our study, more WRKY genes were identified, and in addition to the WRKY domain, other domains such as Plant_zn_clust, TFCD_C superfamily, Uso1_p115_C, Nucleoporin_FG2, BRLZ, and LGIC_ECD were also identified. Moreover, more motifs were detected, and new cis-acting elements such as the wound-responsive element were identified, suggesting that the corresponding genes may have more novel functions.In this study, the functions of some poplar WRKY genes were predicted by comparing with orthologous genes in well-established model plants (*Arabidopsis thaliana*) and rice. For example, the ortholog of PtWRKY16, AT2G44745.1, negatively regulates Fe transport into seeds and inhibits GSH1 expression to negatively regulate cadmium tolerance in *Arabidopsis*. The ortholog of PtWRKY31, AtWRKY65 (AT1G29280.1), and the ortholog of PtWRKY81, AtWRKY31 (AT4G22070.1), can actively respond to toxicity from cadmium and cesium, respectively. AtWRKY13 (AT4G39410.1) can enhance cadmium tolerance by promoting the production of D-cysteine desulfhydrase and hydrogen sulfide. AtWRKY33 (AT2G38470.1) plays a crucial role in preventing metal toxicity in *Arabidopsis* by timely blocking Cd uptake. AtWRKY45 (AT3G01970.1) positively regulates cadmium tolerance in *Arabidopsis* by activating the expression of PCS1 and PCS2, and its corresponding orthologs, PtWRKY37, PtWRKY87, and PtWRKY66, may also confer cadmium tolerance. These new findings reveal the functional roles of WRKY genes in poplars' adaptation to cadmium stress, which were not covered in Chen et al.'s study. The above content is also presented in the discussion, line 583.

2. **Response to comment:**

Experimental results are mostly exploratory or weak in relation to the relevance of the selected PtWRKY95 gene, which is only tested in an heterologous system without a clear relevance for the role in using poplar as a Cd accumulator in contaminated settings. Not much study of this gene in poplar is performed beyond gene expression.

**Response:**

Thank you for your careful work. This study mainly aimed to screen cadmium resistant genes in poplar through RNA-seq and conduct preliminary functional verification of key WRKY genes. The results showed that under cadmium stress, PsnWRKY95 was upregulated by 39.39-fold, 13.14-fold, and 8.62-fold in poplar root, stem, and leaf, respectively. It is precisely because PsnWRKY95 has been rarely studied in poplar that we carried out in-depth research on it. Since poplar, as woody plants, have a large biomass and a long research cycle, we first used the model plant tobacco for preliminary functional verification. Many studies have shown that heterologous research using tobacco is reliable, as reported in the following:

Zhang X, Cheng Z, Fan G, Yao W, Li W, Chen S, Jiang T. Functional analysis of PagNAC045 transcription factor that improves salt and ABA tolerance in transgenic tobacco. BMC Plant Biol. 2022 May 25;22(1):261.

Yao W, Zhang D, Zhou B, Wang J, Li R, Jiang T. Over-expression of poplar NAC15 gene enhances wood formation in transgenic tobacco. BMC Plant Biol. 2020 Jan 8;20(1):12.

Cheng Z, Zhang X, Zhao K, Zhou B, Jiang T. Ectopic expression of a poplar gene NAC13 confers enhanced tolerance to salinity stress in transgenic Nicotiana tabacum. J Plant Res. 2020 Sep;133(5):727-737.

Currently, we are conducting in-depth studies on poplar genetic transformation, functional verification, and regulatory networks of PsnWRKY95, and the results will be published in subsequent work.

3. **Response to comment:**

Phylogeny made with NJ (a very simple and limited inference method) and without bootstrapping or any other means of providing support to the nodes

**Response:**

Thank you for your careful work. We reconstructed the unrooted phylogenetic tree using the maximum likelihood (ML) method with MEGA 7.0 software, employing 1000 bootstrap replications and Poisson correction. The visualization and annotation of the phylogenetic tree were performed using the JTT+G+I model and ITOL (https://itol.embl.de/) software. Additionally, the method description has been revised in Section 2.2.

4. **Response to comment:**

It is not clearly stated why the authors chose the cross *Populus simonii × P. nigra* for several experiments and not P. trichocarpa.

**Response:**

Thank you for your careful work. The geographical context of this study is the alpine mining areas in Heilongjiang. The primary experimental organism is *Populus simonii × P. nigra*. In comparison to other poplar cultivars, this hybrid exhibits rapid growth, a tall and straight stature, robust adaptability, and excellent cold hardiness traits that render it particularly well-suited for cultivation in alpine regions like Heilongjiang, China.Through mass selection of improved varieties, regional trials and years of practice, it has been proven to be an excellent fast-growing and high-yielding tree species. However, the genome of *Populus simonii × P. nigra* is still incomplete and has not been published. As a model plant, *Populus trichocarpa* has a very mature genome after years of sequencing and correction. Moreover, through extensive transcriptome sequencing of *Populus simonii × P. nigra*, we found that the gene sequences of *Populus simonii × P. nigra* and *Populus trichocarpa* are extremely similar with high homology, reaching more than 99%. Therefore, in this study, the *Populus trichocarpa* genome was used as the reference genome to search for genes, and *Populus simonii × P. nigra* was used as the experimental material for gene expression and functional verification experiments. This has been explained in the introduction, line 125.

5. **Response to comment:**

RNA-Seq reads should be deposited in a public repository and an accession to access these data provided.

**Response:**

Thank you for your careful work. We highly recognize the importance of data sharing for research integrity and progress in the field. The raw RNA-seq data involved in this study have been properly stored and subjected to strict quality control and standardized processing. However, since this batch of data is related to the ongoing series of research in our research group (including unpublished gene expression regulatory network analysis), in accordance with the internal data management regulations of the research group and the continuity requirements of related research, it is currently not possible to upload all raw sequencing data to public databases. Nevertheless, we commit that all RNA-seq analysis data of the WRKY family involved in this paper have been uploaded as supplementary files S9 Table in this manuscript, and have also been uploaded to the database with the DOI: 10.6084/m9.figshare.29626553 as confidential archived files for editors and reviewers to check. Thank you for your understanding and support.

6. **Response to comment:**

L65: "modifying plant functional protein to play a miraculous role", please provide a more adequate adjective than miraculous.

**Response:**

Thank you for your careful work. We have revised "miraculous" to "remarkable".

7. **Response to comment:**

L77: how is that "Many studies have shown that WRKY transcription factors, which can regulate ... are referred to as universal factor." Universal factor?

**Response:**

Thank you for your careful work. We have revised "universal" to "versatile".

8. **Response to comment:**

P12, L153: Please specify the program used to calculate Ka/Ks substitution rates.

**Response:**

Thank you for your careful work. We calculated the Ka/Ks substitution rates using the "Simple Ka/Ks Calculator" function in TBtools software. This tool employs the Nei-Gojobori algorithm to estimate synonymous (Ks) and non-synonymous (Ka) substitution rates by aligning the coding regions of segmental duplication sequences of PtWRKY genes, and all Ka/Ks values are derived from this analysis. The specific information has been described in Section 2.3, line 170.

9. **Response to comment:**

L176: the DESeq package is mentioned for determining DEGs but no details are given for read processing, mapping and gene count calculation.

**Response:**

Thank you for your careful work. After quality assessment of RNA-seq raw data with FastQC, low-quality reads and adapters were removed using Trimmomatic; clean reads were aligned to the *Populus trichocarpa* reference genome via HISAT2; gene-level read counts were generated with featureCounts, followed by differential expression analysis using the DESeq2 package in R (DEGs screened by |FC| ≥ 2 and p-value ≤ 0.05). Gene expression plots were generated in Excel. We have supplemented the relevant content in the research methods of Section 2.5 in line 196.

10. **Response to comment:**

L209: the title "Expression PsnWRKY95 tobacco obtaining and cadmium resistance analysis" is confusing

**Response:**

Thank you for your careful work. We have revised the title to “Generation of *PsnWRKY95*-overexpressing tobacco and cadmium resistance analysis”.

11. **Response to comment:**

L230: please change "clue" to query.

**Response:**

Thank you for your valuable advice. We have revised "clue" to "query".

12. **Response to comment:**

Figures 8 and 9 have too much white space and is hard to read without zooming in considerably.

**Response:**

Thank you for your valuable advice. We have revised Fig 8 and Fig 9.

13. **Response to comment:**

L398: pĺease clarify here, not only in the figure legend, that STINGS was used to say that " PsnWRKY95 (Potri.018G019700.1) can interact with eight WRKY family proteins..."

**Response:**

Thank you for your careful work. This part of the content has been revised in Section 3.7.

14. **Response to comment:**

Figure 10C: nuclear localization is expected and should be a supplementary figure.

**Response:**

Thank you for your valuable advice. We have presented Fig. 10C as Supplementary Material S1 Fig.

15. **Response to comment:**

L416: please correct "Numerous studies have confirmed that the Cd resistant cis-acting element G-box can specifically bind to target genes". The element does not bind target genes.

**Response:**

Thank you for your careful work. We have corrected this expression to “Numerous studies have confirmed that plant transcription factors can specifically bind to the cis-acting element G-box in the promoters of downstream target genes, thereby regulating the downstream target genes and exerting cadmium resistance function” in Section 3.8.
